# A multi-parametric workflow for the prioritization of mitochondrial DNA variants of clinical interest

**DOI:** 10.1007/s00439-015-1615-9

**Published:** 2015-11-30

**Authors:** Mariangela Santorsola, Claudia Calabrese, Giulia Girolimetti, Maria Angela Diroma, Giuseppe Gasparre, Marcella Attimonelli

**Affiliations:** Department of Biosciences, Biotechnologies and Biopharmaceutics, University of Bari, Via E.Orabona 4, 70126 Bari, Italy; Department of Science and Technologies, University of Sannio, Via Port’Arsa 11, 82100 Benevento, Italy; Department of Medical and Surgical Sciences, Medical Genetics, University of Bologna Medical School, via Massarenti 9, 40138 Bologna, Italy

## Abstract

Assigning a pathogenic role to mitochondrial DNA (mtDNA) variants and unveiling the potential involvement of the mitochondrial genome in diseases are challenging tasks in human medicine. Assuming that rare variants are more likely to be damaging, we designed a phylogeny-based prioritization workflow to obtain a reliable pool of candidate variants for further investigations. The prioritization workflow relies on an exhaustive functional annotation through the mtDNA extraction pipeline MToolBox and includes Macro Haplogroup Consensus Sequences to filter out fixed evolutionary variants and report rare or private variants, the nucleotide variability as reported in HmtDB and the disease score based on several predictors of pathogenicity for non-synonymous variants. Cutoffs for both the disease score as well as for the nucleotide variability index were established with the aim to discriminate sequence variants contributing to defective phenotypes. The workflow was validated on mitochondrial sequences from Leber’s Hereditary Optic Neuropathy affected individuals, successfully identifying 23 variants including the majority of the known causative ones. The application of the prioritization workflow to cancer datasets allowed to trim down the number of candidate for subsequent functional analyses, unveiling among these a high percentage of somatic variants. Prioritization criteria were implemented in both standalone (http://sourceforge.net/projects/mtoolbox/) and web version (https://mseqdr.org/mtoolbox.php) of MToolBox.

**Electronic supplementary material:**

The online version of this article (doi:10.1007/s00439-015-1615-9) contains supplementary material, which is available to authorized users.

## Introduction

The exponential growth of human mitochondrial DNA (mtDNA) sequences available in public databases (Brandon et al. 2005; van Oven and Kayser 2009; Rubino et al. 2012) is probably the best current hallmark of the central role the mitochondrial genome plays in medicine, forensics and anthropology. In particular, clinicians have recently re-discovered the ‘neglected genome’ (Pesole et al. 2012) as a pivotal determinant or modifier of an increasing number of pathologies, including Alzheimer (Adeghate et al. 2013), cancer (Verschoor et al. 2013), diabetes (Patti and Corvera 2010; Mercader et al. 2012; Adeghate et al. 2013), spinocerebellar ataxias (Mancini et al. 2013) and several types of sclerosis (Patti and Corvera 2010). Because the role of mtDNA variants in most of these phenotypes is still a matter of debate, it is fundamental that either novel or previously reported mtDNA variants of interest are highlighted and brought forward to subsequent analyses, since functional studies aimed at ascertaining the potential pathogenicity often require cumbersome efforts. After sequencing and assembling an mtDNA genome, the first analytical step is usually the identification of positions differing from the chosen reference sequence. Next, with the aim of identifying variants of potential interest for a disease or a particular phenotype, such positions should be further filtered after determining the correct genetic background (haplogroup), so that fixed evolutionary allelic variants may be promptly recognized. Indeed, human mitochondrial phylogeny is described by haplogroup classification based on clusters of closely related evolutionary haplotypes, defined by the pattern of genetic markers occurring in the entire mtDNA and reflecting the migration of human populations over continents (Watson et al. 1997; Balter 2011).

Although clinicians are seldom familiar with the complexity of haplogroups, evolutionary and adaptive aspects should not be ignored in clinical studies, whereby the genetic association between haplogroup-defining variants and clinical phenotypes has been traced (Ghelli et al. 2009; Khan et al. 2013; Peng et al. 2013; Zhang et al. 2013). However, in the search for clinically relevant mtDNA variants, it is useful to rule out those evolutionary fixed, thus limiting variability analyses to few variants, annotated with estimation of conservation and prediction of pathogenicity, to obtain a shortlist of candidates that affect function of the gene/protein. Also, the heteroplasmic fraction of a sequence variant, whenever available, may not be neglected. Indeed, the advent of high-throughput sequencing technologies in mitochondrial genetics has revealed that a wide range of mtDNA variants at low heteroplasmy occurs also in healthy individuals (Payne et al. 2013; Diroma et al. 2014), and varies among tissues (He et al. 2010).

Overall, there is an urgent need for a common workflow based on stringent criteria, to be implemented by researchers who face the challenge of in-depth analysis of mtDNA sequences, which would allow them to recognize the influence of few variants on the phenotype, thus facilitating the functional assay.

In this paper, we propose and validate a workflow for prioritizing functionally important non-synonymous variants, starting from the variant annotation process already implemented in MToolBox based on the use of the Macro Haplogroup Consensus Sequences (MHCS) (Calabrese et al. 2014) and taking into account pathogenicity predictors. A nucleotide variability cutoff and a disease score threshold are established, to prioritize a pool of candidate variants affecting function, which may then be further investigated. Disease scores and prioritization criteria are now implemented in both the standalone version (http://sourceforge.net/projects/mtoolbox/) as well as in the web version of MToolBox at MSeqDR portal (https://mseqdr.org/mtoolbox.php) (Falk et al. 2014).

## Materials and methods

### The MToolBox variant annotation process

MtDNA variants identified by the MToolBox pipeline (Calabrese et al. 2014) are thoroughly parsed through an annotation process which is mainly based on the comparison with both the two widely used rCRS and RSRS reference sequences and the recognition of alleles that are not shared with the sample-specific MHCS. MHCSs, integrated in the MToolBox package, were generated from 32 multiple alignments of complete mitochondrial sequences from 14,144 healthy individuals available in HmtDB (November 2013 update) belonging to the 32 chosen macro-haplogroups. Each macro-haplogroup-specific multi-alignment was then subjected to nucleotide composition analysis by applying the SiteVar algorithm (Pesole and Saccone 2001) to determine the allele occurring most frequently in each position thus generating the MHCSs.

The annotations provided by MToolBox for each variant include:

- nucleotide site-specific variability estimated on the multi-alignment of the updated healthy genomes reported in HmtDB;
- predictions of pathogenicity for non-synonymous variants by applying MutPred (Li et al. 2009), HumDiv- and HumVar-trained PolyPhen-2 models (Adzhubei et al. 2013), SNPs&GO, PhD-SNP (Capriotti et al. 2013), and PANTHER algorithms (Thomas and Kejariwal 2004). Each predictor assigns to each sequence variant a probability score of pathogenicity as well as a qualitative prediction ‘disease’, ‘neutral’ or ‘unclassified’.
- Mitomap (Lott et al. 2013) annotations referring to disease-associated mutations, occurring in coding and control regions and somatic mutations together with their state of homoplasmy/heteroplasmy;
- links to OMIM (http://omim.org).

All these data are available in the ‘patho_table’, a tab delimited file provided by the MToolBox package, listing all possible 24,195 non-synonymous nucleotide substitutions which may occur within the 13 human mitochondrial protein encoding genes, as previously reported (https://sourceforge.net/projects/mtoolbox/; Pereira et al. 2011). Links to Mamit-tRNA (Pütz et al. 2007) web resources were added to provide the user with a general view of variants localization within the mitochondrial tRNA sequence structure.

### Macro-haplogroup consensus sequences (MHCS) phylogeny

The robustness of 32 MHCSs was tested by generating a phylogenetic tree including all MHCSs and two complete mitochondrial sequences, for each macro-haplogroup, derived from Phylotree (van Oven and Kayser 2009). Those sequences are also available in the Human mitochondrial Data Base (HmtDB—Rubino et al. 2012) which reports all publicly available human mitochondrial genomes. Genomes associated to population studies are stored and analyzed as ‘healthy’; genomes from subjects affected by mitochondriopathies are reported in a separate category and annotated as ‘patient’. All data required to establish if an mtDNA genome sequence belongs to the ‘healthy’ category are obtained from the GenBank entry, papers and upon request to the authors. In addition, the multi-alignment and its manual editing allow to check the quality of sequences to detect any sequencing errors.

The MHCS phylogenetic tree was produced according to the Maximum Likelihood method, based on the Jukes–Cantor substitution model (Jukes and Cantor 1969). The quality of the tree topology was assessed by bootstrap analysis of 500 replicates (Felsenstein 1985). Analyses were performed through the functions implemented in MEGA5 software (Hall 2013).

### Datasets

The prioritization workflow was validated on a dataset of 125 mtDNA genomes belonging to individuals affected by Leber’s Hereditary Optic Neuropathy (LHON), sequenced by Sanger technology and stored in HmtDB (http://www.hmtdb.uniba.it) (Rubino et al. 2012) (Supplementary Table 1—SampleData). This dataset was chosen to evaluate the performance of this workflow in identifying the known LHON-causative mutations since 42 % of these genomes was expected to harbor at least one of the primary mutations included in the panel of the ‘Top 14 LHON’ annotated in Mitomap (Lott et al. 2013).

The efficiency of the workflow to prioritize and recognize tumor-specific variants with a functional impact was also tested on mtDNA sequences obtained from 20 ovarian cancer samples, collected within a concluded clinical study, at S.Orsola-Malpighi Hospital, Bologna, Italy, during the period 2012–2013. Informed consent had been obtained in compliance with the Helsinki Declaration and the study had been approved by the local ethical committee. DNA was available also from the corresponding non-tumor tissue of patients and was used in the context of this study to test the germline nature of identified variants. List of specimens and HmtDB identifiers, obtained after submission of sequences to the HmtDB, are reported in Supplementary Table 2—SampleData. Sanger sequencing of the whole mtDNA was performed as previously described (Kurelac et al. 2013), to prevent nuclear mitochondrial sequence (NumtS) (Simone et al. 2011) co-amplification. The somatic (tumor-specific) nature of mitochondrial variants was ascertained by sequencing mtDNA from matched non-tumor tissue and validated on a second PCR product.

Finally, to test our workflow also on data generated from high-throughput technologies, Whole Exome Sequencing (WXS) BAM files (Binary Alignment/Map) from 90 matched samples (primary solid tumor/peripheral blood) from Colorectal Adenocarcinoma (COAD) patients were downloaded from the online repository of the consortium dbGaP (https://cghub.ucsc.edu/). These data were generated from the Baylor College of Medicine (BMC) center of The Cancer Genome Atlas (TCGA, http://cancergenome.nih.gov/) on Illumina platform and mapped on GRCh37-lite reference (Genome Reference Consortium Human Build 37, accession = “GCA_000001405.1”). The samples featuring a mean read depth >10X across the mitochondrial genome after its extraction through MToolBox were brought forward in the analysis (Supplementary Table 3—BloodSamples and TumorSamples sheets).

### Denaturing high-performance liquid chromatography (dHPLC) analysis on OC samples

For the variants found in Ovarian Cancer (OC) samples, PCR was performed using AmpliTaq Gold polymerase (Applied Biosystems). For m.3380G>A in *MT*-*ND1* fw-5′-ATACCCACACCCACCCAAGA-3′ and rv-5′-AGATGTGGCGGGTTTTAGGG-3′ primers were used, for m.9837G>A in *MT*-*CO3* fw-5′-TCAATCACCTGAGCTCACCA-3′ and rv-5′-ACCACATCTACAAAATGCCAGT-3′ primers were used, for m.14969T>A in *MT*-*CYB* fw-5′-AACTTCGGCTCACTCCTTGG-3′ and rv-5′-TCACGGGAGGACATAGCCTA-3′ primers were used. The amplification product was analyzed by WAVE Nucleic Acid Fragment Analysis System (Transgenomic, Omaha, NE, USA). Data analysis was performed as previously described (Frueh and Noyer-Weidner 2003; Kurelac et al. 2012).

### Mitochondrial DNA extraction, variant detection and annotation

MToolBox pipeline (Calabrese et al. 2014), including several steps as read mapping and NumtS filtering, post-mapping processing, genome assembly, haplogroup prediction and variant annotation, was used to extract the off-target mitochondrial genomes from the WXS COAD BAM files obtained from the TCGA repository, and then to annotate each variant allele. Fasta files from LHON and ovarian cancer samples were also used as input for MToolBox to annotate mitochondrial variants and related features.

### Disease Score definition for non-synonymous variants

A training dataset of 53 mtDNA non-synonymous variants (Table 1; Supplementary Table 4), previously validated as affecting function, including 28 disease-associated mutations annotated in Mitomap as ‘confirmed’ to be pathogenic by two or more independent laboratories (Lott et al. 2013), and 25 clearly pathogenic cancer-associated mutations (Gasparre et al. 2007; Porcelli et al. 2010; Pereira et al. 2012), was used to define the ‘disease score’ (as described in the “Results” section ‘Disease Score definition’) of any non-synonymous mtDNA variants, by weighting the 6 above-listed pathogenicity predictions (Thomas and Kejariwal 2004; Adzhubei et al. 2013; Capriotti et al. 2013) available in the ‘patho_table’ implemented in MToolBox (https://sourceforge.net/projects/mtoolbox/). These six methods were chosen among the most widely used pathogenicity predictors, available online for a fast evaluation of large-scale data from sequencing, although their often-contradictory predictions demand a way to weigh their reliability. More details regarding the features used by any method to predict the impact of amino acid allelic variants on protein structure/function are available in (Thomas and Kejariwal 2004; Adzhubei et al. 2013; Capriotti et al. 2013).

**Table 1:** List of 53 non-synonymous variants composing the training dataset

Non-synonymous variant	Locus	Non-synonymous variants	Locus	Non-synonymous variants	Locus
T9185C	MT-ATP6	T3931C	MT-ND1	G10573A	MT-ND4L
T9176C	MT-ATP6	G3392A	MT-ND1	G13042A	MT-ND5
T9176G	MT-ATP6	G3733A	MT-ND1	T12706C	MT-ND5
G8839A	MT-ATP6	T3949C	MT-ND1	T13540C	MT-ND5
T8993C	MT-ATP6	G3697A	MT-ND1	G13513A	MT-ND5
T8993G	MT-ATP6	T3679C	MT-ND1	A13514G	MT-ND5
C6567T	MT-CO1	G3922A	MT-ND1	G13178A	MT-ND5
T6210C	MT-CO1	G4831A	MT-ND2	T12797C	MT-ND5
T15843C	MT-CYB	G4975A	MT-ND2	T13847C	MT-ND5
T15813G	MT-CYB	T10158C	MT-ND3	T13271C	MT-ND5
T15209C	MT-CYB	T10191C	MT-ND3	C14568T	MT-ND6
G3700A	MT-ND1	G10197A	MT-ND3	C14482A	MT-ND6
C4171A	MT-ND1	G12056A	MT-ND4	C14482G	MT-ND6
G3460A	MT-ND1	T11613C	MT-ND4	T14484C	MT-ND6
T4222C	MT-ND1	C11777A	MT-ND4	A14495G	MT-ND6
G3635A	MT-ND1	G11778A	MT-ND4	G14459A	MT-ND6
G4148A	MT-ND1	G11475A	MT-ND4	T14487C	MT-ND6
G3890A	MT-ND1	T10663C	MT-ND4L		

The table lists non-synonymous variants and related locus previously validated as affecting the protein function and included in the training dataset used to define the disease score

### Disease Score threshold

To derive the Disease Score Threshold (DST) for assessing the potential functional impact of the non-synonymous variants, the mixture model of two normal distributions (McLachlan and Peel 2000) was fitted to the disease scores related to 1872 non-synonymous variants observed in 15,385 mtDNA genomes from healthy individuals, carefully selected as complete sequences (http://webservice.cloud.ba.infn.it/hmtdb/HmtDBHealthyGenomes_References.xlsx) and stored in HmtDB (May 2014, Rubino et al. 2012). It was not possible to define a DST from non-synonymous variants found in mtDNA genomes from patients, as stored in HmtDB. This dataset suffered a sampling bias, in which certain pathologies (as LHON, Alzheimer’ disease, etc.) were over-represented, while others (as MELAS, etc.) were under-represented. The analysis was performed using the normalmixEM function from the mixtools package (Benaglia et al. 2009) implemented in R version 3.1.1.

## Results

### Macro-haplogroup consensus sequences reliability

Based on the hypothesis that the mtDNA variants spread and fixed in one or more populations (e.g., haplogroup-defining variants) may be modifiers, but are less likely to be causative of a pathologic phenotype, the identification and prioritization of potentially pathogenic variants require retaining the rare ones, more prone to affect the gene/protein function (i.e., featuring high pathogenicity scores and occurring in highly conserved sites subjected to functional and selective constraints). In this perspective, comparing an mtDNA sequence to the related haplogroup-specific MHCS (Calabrese et al. 2014) may facilitate the process of prioritization by filtering out the fixed evolutionary variants and recognizing the rare ones. The consistency of MHCSs as consensus is supported by the clustering of such sequences with real genomes belonging to the same haplogroup, as shown in the Supplementary Fig. 1. Hence, the simultaneous recognition of any variant with respect to rCRS (Andrews et al. 1999), RSRS (Behar et al. 2012) and MHCS was designed as the first step of the prioritization process to obtain a reliable pool of candidate variants, which may warrant further investigations. The 32 MHCSs will be updated according to the availability of new mtDNA sequences in the HmtDB database.

### Disease Score definition

A high number of mtDNA variants annotated in the literature as ‘affecting function’ are non-synonymous mutations which involve the 13 protein-coding genes (Achilli et al. 2012; Pereira et al. 2012), although high is also the number of mutations leading to functional damages in tRNA, rRNA and *MT-DLOOP* loci. The need to estimate the potential functional impact of any non-synonymous variant is hence a priority and yet the discrepancy of their pathogenicity predictions (Thomas and Kejariwal 2004; Adzhubei et al. 2013; Capriotti et al. 2013), when different algorithms are used, highlights the need for methods that may weigh the reliability of predictors and yield a single pathogenicity score. A disease score was, therefore, defined based on the weighted mean of the probabilities that an amino acid substitution may affect gene/protein function, as provided by each pathogenicity predictor. This score ranges between 0 and 1. The weight for each *i*th pathogenicity predictor was calculated as:

$$W_{i} = ({\text{hp}}_{i} + {\text{cp}}_{i} )/2n$$

where hp_*i*_ is the number of times the *i*th predictor out of six provides the highest probability for each training mutation (see “Materials and methods” section) to affect function, cp_*i*_ is the number of times the *i*th algorithm performs a correct prediction confirming the functional impact of the previously validated mutations (Lott et al. 2013), and *n* is the number of mutations used for the training (Supplementary Table 4—Training dataset). HumDiv- and HumVar-trained PolyPhen-2 models (Adzhubei et al. 2013) were the most effective predictors for the training non-synonymous mutations based on the established weights. The Disease Score (DS) of a non-synonymous variant was then calculated as reported in “Box 1”.

### Box 1: Disease score (DS)

$${\text{DS}} = \frac{{({\text{MPp}} \times {\text{MPw}}) + ({\text{PPDp}} \times {\text{PPDw}}) + ({\text{PPVp}} \times {\text{PPVw}}) + ({\text{PTp}} \times {\text{PTw}}) + ({\text{PSp}} \times {\text{PSw}}) + ({\text{SGp}} \times {\text{SGw}}) }}{{{\text{MPw}} + {\text{PPDw}} + {\text{PPVw}} + {\text{PTw}} + {\text{PSw}} + {\text{SGw}}}}$$

where MPp = MutPred probability, PPDp = Polyphen-2 HumDiv probability, PPVp = Polyphen-2 HumVar probability, PTp = PANTHER probability, PSp = PhD-SNP probability, SGp = SNPs&GO probability, MPw = MutPred weight, PPDw = Polyphen-2 HumDiv weight, PPVw = Polyphen-2 HumVar weight, PTw = PANTHER weight, PSw = PhD-SNP weight, SGw = SNPs&GO weight.

### Prioritization criteria of mtDNA non-synonymous variants

**Fig. 1:**
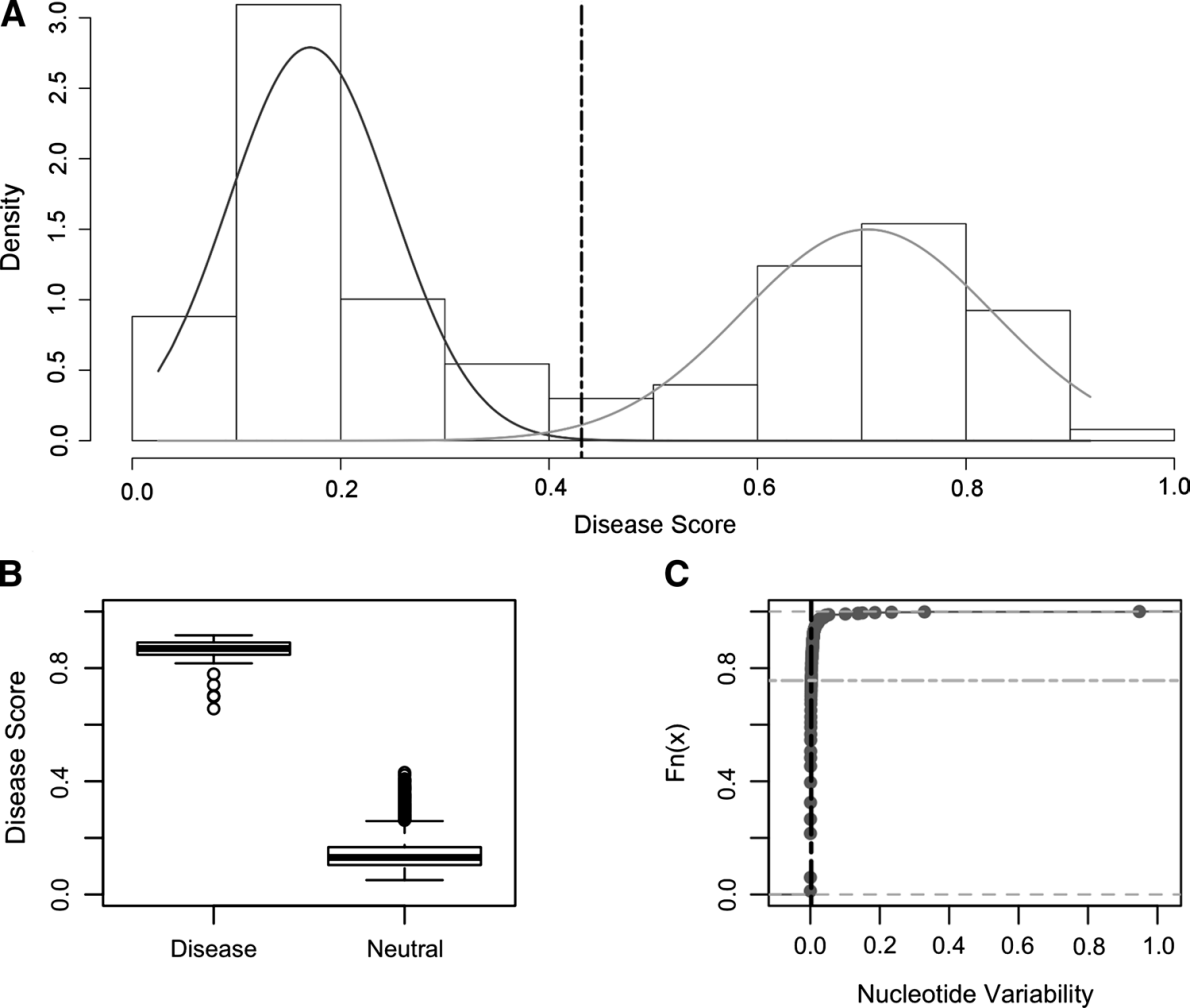
**a** The histogram graphs the bimodal distribution of disease scores associated to 1872 non-synonymous variants (HmtDB, May 2014) observed in mtDNA sequences from healthy individuals and stored in HmtDB. The *solid lines* indicate the two gaussian components of the mixture model (McLachlan and Peel 2000) (46 and 54 %, respectively). The first component of the mixture model with the lowest disease score values included the most benign non-synonymous variants. The *vertical dashed line* is drawn at the selected Disease Score Threshold, DST, defined as 0.4311; non-synonymous variants featuring a DS above 0.4311 may, therefore, be considered potentially affecting function. **b** *Box-plot diagram* shows the disease scores of non-synonymous variants by class of ‘Neutral’ or ‘Disease’ prediction (disease scores ranging from 0.05 to 0.4311 and from 0.6565 to 0.9162, respectively, for each class) as returned by all six pathogenicity predictors implemented in MToolBox. *Circles* represent the outliers. **c** Empirical cumulative distribution function of nucleotide variability associated with the 816 non-synonymous variants, featuring a disease score above the established DST. *Dashes vertical line* indicates the nucleotide variability cutoff, NVC = 0.0026, defined as the third quartile of such distribution. Non-synonymous variants showing variability values below the NVC are filtered by the variant prioritization workflow

A Disease Score Threshold (DST) and a nucleotide variability cutoff (NVC) were then established to discriminate non-synonymous mtDNA variants contributing to a potential defective phenotype among the candidate rare ones. The distribution of DSs (as described in “Box 1”) for the non-synonymous variants observed in the mtDNA genomes from healthy individuals (see “Materials and methods” section), highlighted a mixture of two normal components (McLachlan and Peel 2000) shown in the histogram (Fig. 1a). It was assumed that the second component of the mixture model with the highest disease score values potentially included the most damaging variants, since the DS of variants predicted by all pathogenicity predictors as ‘neutral’ or ‘disease’ did not overlap (Fig. 1b). The DST was chosen as the lowest DS value by which the probability to belong to the second mixture model component is ten times greater than the probability to belong to the first one (0.4311—Fig. 1a). Variants showing a DS above 0.4311 may, therefore, be considered potentially functional. Moreover, an additional DST was also calculated on disease scores distribution of ‘all possible non-synonymous variants’, as reported in the patho-table provided with MToolBox (Calabrese et al. 2014). A bimodal distribution of the DSs, similarly to that for non-synonymous variants observed in healthy individuals, was obtained (Supplementary Fig. 2). In this case, however, the second component of the mixture model underlined a higher number of variants, never occurred in any mtDNA sequence, with DSs higher than those from healthy genomes. This very high DST may underestimate the potential functional impact of a variant, since the majority of variants showing a very high DS have never been observed in mtDNA genomes from both healthy and diseased individuals. Furthermore, by taking into account the nucleotide variability values associated to the 816 observed non-synonymous variants featuring disease score above the established DST (Fig. 1c), we chose the third quartile of this distribution as nucleotide variability cutoff (NVC) for variants with a possible functional impact. The resulting NVC was 0.0026. The established cutoffs were applied to the dataset of all possible non-synonymous mitochondrial variants (reported in ‘patho_table’ implemented in Calabrese et al. 2014; https://sourceforge.net/projects/mtoolbox/), selecting the 70.8 % of variants as potentially functional on the strength of both nucleotide variability lower than 0.0026 and disease score higher than 0.4311.

In summary, by taking into account the functional annotation already implemented in MToolBox (Calabrese et al. 2014), the in silico prioritization criteria here proposed are intended to easily target the mtDNA variants that most likely affect the gene/protein function, by prioritizing those non-synonymous variants (1) simultaneously identified by rCRS, RSRS and MHCS, (2) non-haplogroup defining, (3) featuring nucleotide variability lower than NVC and (4) having a disease score above the DST. In addition, variants showing heteroplasmy levels greater than or equal to a user-established value were included among the variants potentially impacting on phenotype. The prioritization process was tested on samples from the three different datasets: a series of LHON mtDNA sequences was used in representation of one of the most widely studied canonical mitochondrial diseases; an ovarian cancer and a COAD dataset were used with the aim to test the workflow for its ability to recognize potentially functional tumor-specific variants starting from both Sanger sequencing and Whole Exome (WXS), respectively.

### LHON-derived samples variant prioritization analysis

LHON is a paradigmatic mitochondrial disorder usually caused by mtDNA point mutations leading to amino acid changes in genes encoding the subunits of complex I of the mitochondrial respiratory chain (Abu-Amero and Bosley 2006). Genes *MT*-*ND1* and *MT*-*ND6* are reported as hotspots for LHON-causative mutations (Reynier et al. 1999; Valentino et al. 2004; Fraser et al. 2010), which harbor ‘14 Top primary mutations’ (Wallace et al. 1988; Howell et al. 1991; Johns et al. 1992; Brown et al. 1995; Chinnery et al. 2001; Kim et al. 2002; Gropman et al. 2004; Achilli et al. 2012) annotated in Mitomap (Lott et al. 2013).

**Fig. 2:**
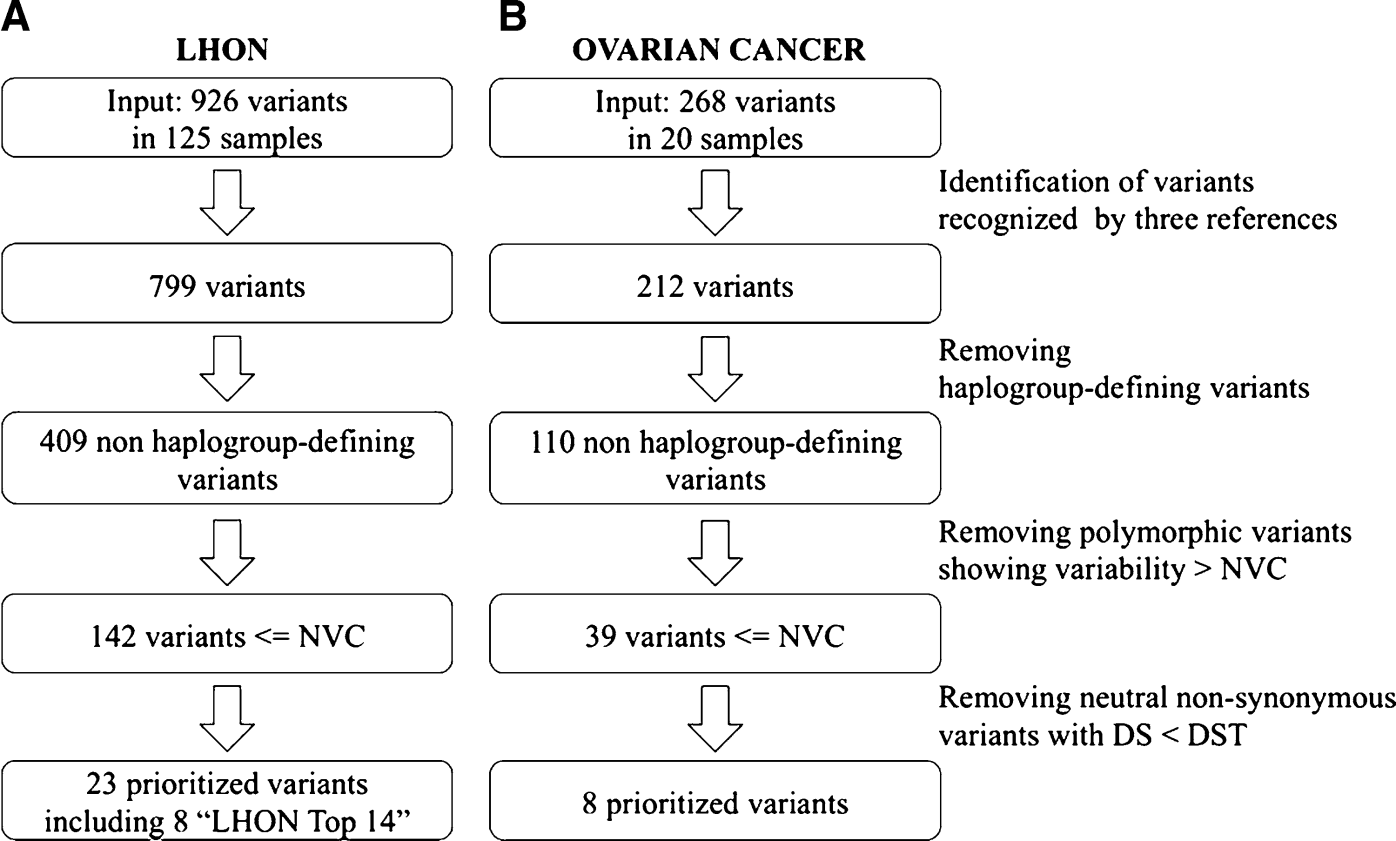
The stepwise prioritization workflow and the related number of mitochondrial variants filtered in any step performed on the full lists of any detected variants annotated in *A* LHON and *B* ovarian datasets from Sanger sequencing

To evaluate the performance of the prioritization criteria in identifying the known causative LHON mutations and/or suggesting novel variant candidates for further analyses, the prioritization process was applied to 125 LHON-derived mtDNAs. The workflow is shown in Fig. 2a. We identified 926 variants with an average of 72 variants per genome (Supplementary Table 1). We first focused on the subset of variants recognized by rCRS, RSRS and MCHS and occurring in non-haplogroup-defining sites. Among these, only 142 variants were brought forward in the analysis since they occurred in positions subjected to functional constraint and then featuring variability values lower than or equal to the NVC value. Next, we filtered the variants mapping on the protein-coding regions and leading to an amino acid change, and evaluated them on the strength of their DS. 23/51 non-synonymous variants (2.5 % of total variants) identified in 66.4 % of 125 analyzed LHON-derived genomes and featuring a disease score above the chosen DST were prioritized as probably affecting function (Fig. 2a). The genomes analyzed harbored 11 out of 14 Top primary LHON mutations; the prioritization process selected 8 out of 11 leaving out m.3700G>A, m.14502T>C and m.14484T>C (Table 2). Specifically, the rare mutation m.3700G>A (*MT*-*ND1*) was not included, as it featured a disease score (DS = 0.35) lower than the chosen DST, suggestive of a benign behavior of the resulting amino acid change on protein function, even though absent in the healthy population (nucleotide variability = 0.00). The mutation m.14502T>C (*MT*-*ND6*) was found in two genomes (PA_EU_DE_0006; PA_AS_CN_0071, the latter showing also the mutation m.14484T>C) and discarded because both DS and NV were outside of the fixed thresholds. Finally, the mutation m.14484T>C (*MT*-*ND6*) found in 95 % of LHON cases was not prioritized due to its NVC higher than the threshold (NV = 0.0046). The list of prioritized variants included also those ones showing the same pathogenicity features of the ‘Top 14 LHON-causative mutations’ (Table 2). Interestingly, they were not previously associated with LHON and, therefore, may warrant further investigation. Furthermore, the multi-parametric workflow was again applied to LHON dataset by removing the filtering step ruling out the haplogroup-defining variants. In that case, the list of prioritized variants counted only two additional variants: m.14337C>T and m.15038A>G, respectively, defining the sample-specific M10a1a1 and B4d1 haplogroups, simultaneously recognized by the three references and featuring variability values lower than NVC and disease scores above the established DST, but not annotated in Mitomap.

**Table 2:** List of prioritized non-synonymous variants in LHON samples

No. samples	Variant allele	Locus	Nt Var	AA change	AA Var	Disease score	Mitomap	1000 genomes
1	10747A	MT-ND4L	0.0000	L93Q	0	0.8781		
1	6448A	MT-CO1	0.0000	P182H	0.0026	0.8325		
1	7042C	MT-CO1	0.0000	V380A	0.0047	0.8044		
1	15156G	MT-CYB	0.0003	Q137R	0.0005	0.9044		
1	7632C	MT-CO2	0.0003	I16T	0.0018	0.4579		
1	9104C	MT-ATP6	0.0007	F193S	0.0075	0.5168		
1	14249A	MT-ND6	0.0016	A142 V	0.0121	0.4498		
1	8551C	MT-ATP6	0.0018	F9L	0.0042	0.7620		0.0008
1	3890A	MT-ND1	0.0000	R195Q	0	0.8184	PE/LS/OA	
2	3733A	MT-ND1	0.0000	E143 K	0	0.8360	LHON Top 14	
9	3635A	MT-ND1	0.0000	S110 N	0	0.7977	LHON Top 14	
1	3733C	MT-ND1	0.0000	E143Q	0	0.8677	LHON	
1	3922A	MT-ND1	0.0000	E206 K	0	0.8939	Head/neck tumor	
1	14495G	MT-ND6	0.0000	L60S	0	0.8616	LHON Top 14	
1	10663C	MT-ND4L	0.0000	V65A	0	0.5776	LHON Top 14	
1	14841G	MT-CYB	0.0000	N32S	0	0.8360	LHON helper mut.	0.0012
1	9655A	MT-CO3	0.0005	S150 N	0.0029	0.7259	Thyroid tumor	0.0008
1	14459A	MT-ND6	0.0006	A72 V	0.0183	0.8655	LDYT/LS/LHON Top 14	0.0008
4	14568T	MT-ND6	0.0009	G36S	0.0079	0.7311	LHON Top 14	
15	3460A	MT-ND1	0.0014	A52T	0.0015	0.7629	LHON Top 14 (95 %)	
2	4171A	MT-ND1	0.0016	L289 M	0.0107	0.6809	LHON Top 14	
2	14482A	MT-ND6	0.0024	M64I	0.0333	0.7923	LHON Top 14	
41	11778A	MT-ND4	0.0025	R340H	0.0516	0.8534	LHON Top 14 (95 %)/PDY	0.0004

Non-synonymous variant recognized on LHON-derived mtDNAs and prioritized according to the established criteria. Number of samples harboring the variant allele (No. samples), mtDNA locus (locus), site-specific nucleotide variability value (Nt Var), amino acid change and variability (AA change and AA Var, respectively), Disease Score, annotations from Mitomap (Lott et al. 2013) and frequencies in 1000 genomes [as implemented in (Calabrese et al. 2014)] are associated with each variant allele. Frameshifts and Premature stop codons are also reported in ‘AA change’ field. None of the LHON variants are involved in haplogroup assignment. For full variants in LHON samples, see Supplementary Table 1

*LDYT* Leber’s hereditary optic neuropathy and Dystonia, *LS* Leigh syndrome, *OA* optic atrophy, *PE* progressive encephalomyopathy, *PDY* progressive dystonia

### Identification of somatic variants in ovarian cancer

Mitochondrial variants have been frequently detected in cancer (Brandon et al. 2006; Jandova et al. 2012), likely as secondary modifier mutations, or as indirect consequences of driver mutations in nuclear genes (Schon et al. 2012). We next sought to determine whether our workflow allows recognizing variants in cancer samples. We started from the assumption that cancer cells are prone to acquire mtDNA variants due to their continuous replication (Coller et al. 2001). Moreover, most often only cancer cells are able to cope with highly pathogenic mtDNA mutations, since they have a deranged energy metabolism (Brandon et al. 2006; Iommarini et al. 2014). One of the problems encountered in assessing mtDNA mutations in cancer is the need to define whether such variants are truly somatic, in which case they may be inferred to be modifiers of tumor progression. We hence attempted to test whether our workflow was able to highlight candidate variants for being somatic, that only transformed cells may withstand in the context of a deregulated cell metabolism. This would avoid sequencing of the whole mtDNA of the non-cancer tissue, which is seldom available particularly for cancers whose nature does not allow resection of surrounding normal tissue (e.g., glioblastoma). To this aim, we exploited 20 mtDNA sequences we had obtained in the context of a clinical study on ovarian cancer, derived from 20 different ovarian carcinomas. For each of these samples, dissected tumor and normal peritumoral tissue were available. Implementation of the workflow on the 20 cancer mtDNAs revealed 268 variants (Supplementary Table 2), with an average of 65.2 variants per sample. 212/268 variants were flagged by all three reference sequences, namely rCRS, RSRS and MCHS, and were brought forward in the analysis. Of these, 110 mapped in non-haplogroup-defining sites, and were further brought forward. 39/110 variants featured a variability value below the NVC. We focused on variants in protein-coding regions since predictions of pathogenicity were available exclusively for non-synonymous variants. 28 of the 39 variants mapped in protein-coding regions. Of the latter, 14 were silent and 13 were non-synonymous. The steps of the prioritization workflow are shown in Fig. 2b. Since we could not rule out a partial contamination by non-tumor stromal cells, which may lead to underestimation of the heteroplasmy fraction, we decided to apply a 0.50 cutoff criterion, upon estimation of heteroplasmy based on the ratio of peaks height on the electropherograms. Overall, 8/13 non-synonymous variants featuring a disease score above the DST, and the frameshift event m.6691insA were finally included in the list of candidate variants for being somatic (Table 3). We next verified whether the prioritized variants were indeed somatic (cancer-specific). Sequencing of the specific amplicon including each of the 9 prioritized variants starting from non-tumor tissues of the corresponding samples revealed that 7/9 variants (78 %) were absent in the non-tumor tissue and, therefore, cancer-associated, whereas 2/9 non-synonymous variants were also detected in non-tumor specimens (22 %) (Table 3). A further DHPLC analysis for the detection of low heteroplasmy (down to 2 %—Kurelac et al. 2012) randomly performed on three cancer-associated variants, revealed that all three were indeed absent from the normal tissue, confirming with a much more sensitive method than Sanger sequencing their true somatic nature (Supplementary Fig. 3). Interestingly, the two non-somatic variants among the prioritized ones were those featuring the lowest DS and the highest NV, suggesting our criteria for inclusion could be made even more stringent. As a final check, we also specifically sequenced the 14 silent variants in the non-tumor specimens from corresponding cancer samples and they all resulted germline, indicating that the NV parameter is necessary but not sufficient to infer somaticity. The list of prioritized variants obtained performing the whole multi-step workflow on this dataset did not differ from that obtained by removing the haplogroup-filtering step.

**Table 3:** List of prioritized mtDNA non-synonymous variants in ovarian cancer samples

Sample	Variant allele	HF	Locus	Nt Var	AA change	Tumor-specific	AA Var	Disease score	1000 genomes
EOC5	3380A	0.8	MT-ND1	0.0003	R25Q	+	0.00	0.8764	0.0004
EOC40	14969C	0.5	MT-CYB	0.0003	Y75H	+	0.00	0.8526	0.0004
EOC16	9837A	0.5	MT-CO3	0.0000	G211S	+	0.00	0.8379	0.0004
EOC20	15255C	0.8	MT-CYB	0.0000	V170A	+	0.00	0.8195	0.0004
EOC20	10696T	0.8	MT-ND4L	0.0000	A76 V	+	0.01	0.7810	
EOC14	6121C	0.5	MT-CO1	0.0007	I73T	+	0.00	0.7054	0.0004
EOC5	8412C	1.0	MT-ATP8	0.0023	M16T	−	0.03	0.6587	0.0008
EOC32	14249A	1.0	MT-ND6	0.0020	A142 V	−	0.02	0.4498	
EOC37	6691.A	0.5	MT-CO1	0	Frameshift	+	0		

Tumor-specific and germline variants recognized on ovarian cancer-derived mtDNAs and prioritized according to the established criteria. Sample identifier (sample), heteroplasmic fraction (HF), mtDNA locus (locus), site-specific nucleotide variability (Nt Var), Amino acid change and variability (AA change and AA Var, respectively), Disease Score, somatic (+) or germline (−) nature (‘tumor-specific/germline’) of variants and frequencies in 1000 genomes [as implemented in (Calabrese et al. 2014)] are associated with each variant allele. For full variants in ovarian cancer samples, see Supplementary Table 2—AllVariants

### Mitochondrial DNA sequences extraction from COAD samples

Numerous studies report somatic mutations in mtDNAs from colorectal cancer patients (Alonso et al. 1997; Polyak et al. 1998; Lièvre et al. 2005; He et al. 2010; Wang et al. 2011) but the correct pattern of COAD-associated mutations has not been yet reconstructed (Skonieczna et al. 2012).

The availability of WXS data obtained by The Cancer Genome Atlas (TCGA) repository provided an extensive characterization of the occurrence of mtDNA variants, also at low heteroplasmy levels, in COAD. Accordingly, we tested the efficiency of our workflow to prioritize a small pool of functionally important variants from the huge amount of data from high-throughput sequencing in this dataset. Pairwise comparison of blood and tumor mtDNA variants was performed to identify germline and tumor-specific mitochondrial variants.

The assembly of mitochondrial WXS reads derived from COAD matched tumor and blood samples showed a mean read depth of 304.86X (median = 258.31X) and 155.88X (median = 127.94X), respectively. The values of mean read depth ranged between 14.90X and 525X in blood and between 13.48X and 1274X in tumor samples. Specifically, the tumor dataset showed a significantly higher read depth (Wilcoxon test, *p* value = 3.194e−06) (Supplementary Table 3—BloodSamples, TumorSamples).

### Tumor-specific variants in COAD samples

**Fig. 3:**
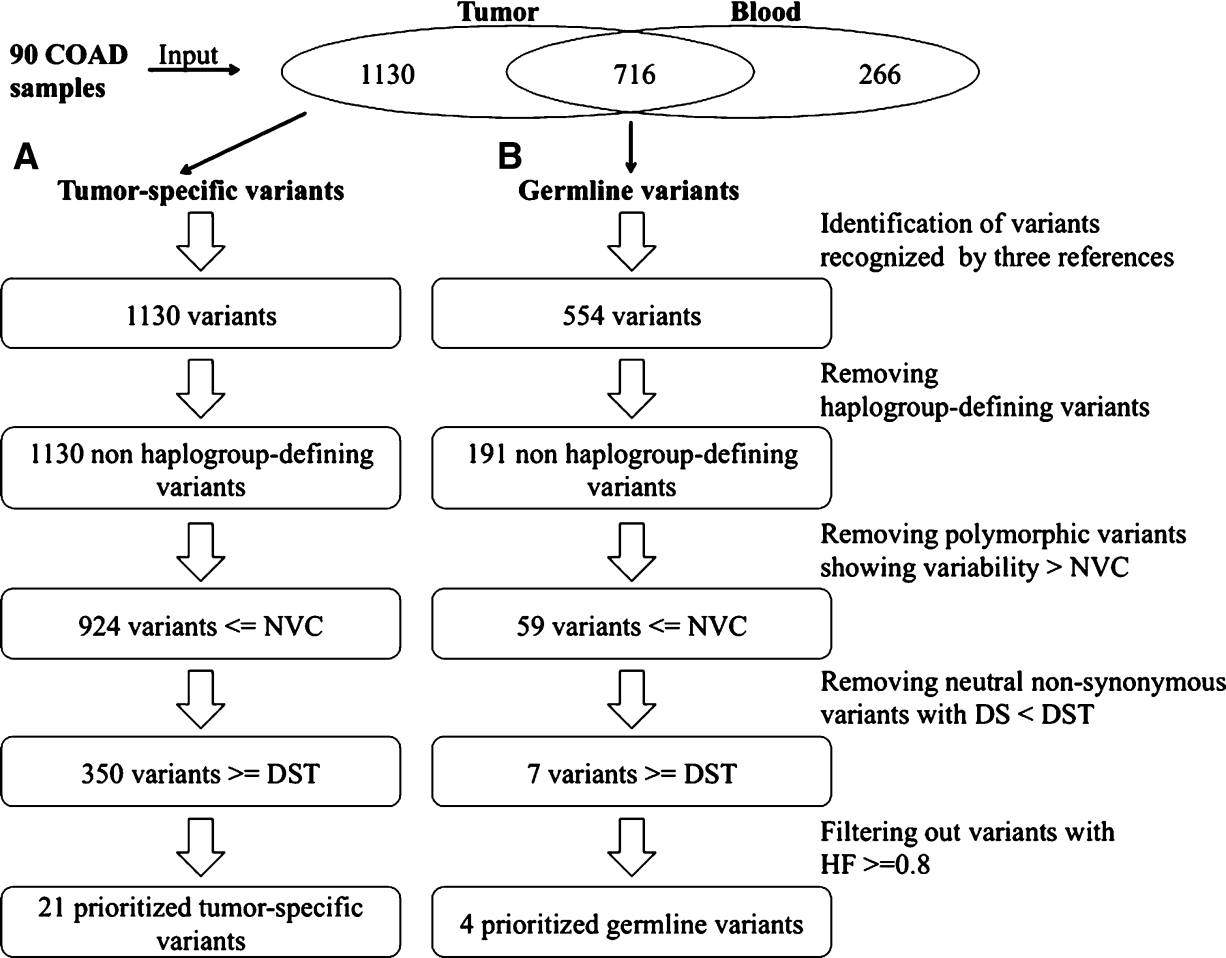
The stepwise prioritization workflow and the related number of mitochondrial variants filtered in any step performed on the full lists of any detected *A* tumor-specific and *B* germline variants annotated in the COAD dataset from Whole Exome Sequencing (WXS). The number of blood-specific variants is also shown

We applied the prioritization process on the full list of tumor-specific variants identified in 86/90 tumor samples (Supplementary Table 2—TumorSpecific). 1130 variants were identified against rCRS, RSRS and MCHS, all mapping on non-haplogroup-defining sites (Fig. 3a). The lack of somatic variants in 4 tumor samples could likely be ascribed to a low mean depth coverage. An average of 14.5 variants per sample was identified. The 82 % of total variants was filtered as featuring a variability value below the chosen NVC. We next focused on the 574 variants mapping in the protein-coding regions. Among these, we identified a subset of 350 non-synonymous variants featuring a disease score above the DST and corresponding to 31 % of all annotated variants. We reasoned that 0.8 would be a reasonable heteroplasmic fraction (HF) cutoff to include potentially affecting function variants with a clear impact on the phenotype, a threshold that is often used as mtDNA variants impact on bioenergetics when 4/5 mtDNA copies are mutated (Chinnery et al. 1997; White et al. 1999; Gasparre et al. 2011; Chinnery and Hudson 2013; Keogh and Chinnery 2013); this additional filtering step drastically reduced the number of candidate non-synonymous variants to 21 (Fig. 3a) and the number of variants leading to premature stop codons to 2. The prioritization criteria were able to prioritize only the 2 % of tumor-specific variants on the 25 % of COAD samples (Table 4). This list included 19 variants not annotated in Mitomap, while the mutations m.3946G>A and m.3380G>A were found associated to Mitochondrial Encephalomyopathy, Lactic Acidosis, and Stroke-like episodes (MELAS), m.15243G>A and m.7623C>T to Hypertrophic Cardiomyopathy (HCM) and LHON, respectively. Finally, the mutation m.14918G>A, found in two samples, was the only one previously associated to colorectal cancer.

**Table 4:** List of tumor-specific non-synonymous variants prioritized in COAD samples

Sample	Variant allele	HF	Locus	Nt Var	AA change	AA Var	Disease score	Mitomap	1000 genomes
A6665101A21D183510	11390A	0.861	MT-ND4	0.0002	Premature Stop Codon	0.01			
A6665201A11D177110	3380A	0.967	MT-ND1	0.0003	R25Q	0.00	0.8800	MELAS	0.0004
AU377901A01D171910	10863A	0.909	MT-ND4	0.0003	S35N	0.00	0.7600		
CM474301A01D171910	14918A	0.814	MT-CYB	0.0003	D58N	0.00	0.7100		0.0004
	14985A	0.881	MT-CYB	0.0000	R80H	0.00	0.9000	Colorectal tumor	0.0017
CM534401A21D171910	11552C	0.819	MT-ND4	0.0000	S265P	0.00	0.8900		
CM586101A01D165010	12814A	0.979	MT-ND5	0.0011	A160T	0.00	0.6900		0.0004
CM586401A01D165010	10854C	0.971	MT-ND4	0.0000	L32P	0.00	0.8500		0.0008
CM616401A11D165010	15243A	0.874	MT-CYB	0.0000	G166E	0.00	0.9000	HCM	0.0004
CM616501A11D165010	3946A	0.935	MT-ND1	0.0001	E214K	0.00	0.9100	MELAS	0.0012
D5653501A11D171910	9645A	0.861	MT-CO3	0.0000	A147T	0.00	0.8100		0.0004
D5654101A11D171910	4810A	0.9	MT-ND2	0.0000	Premature Stop Codon	0.00			
D5693001A11D192410	6798A	0.866	MT-CO1	0.0000	V299M	0.00	0.7700		
DMA0X901A11DA1521	3380A	0.799	MT-ND1	0.0003	R25Q	0.00	0.8800	MELAS	0.0004
	9790T	0.949	MT-CO3	0.0000	S195L	0.00	0.8300		
DMA1D001A11DA15210	4537A	0.925	MT-ND2	0.0000	S23N	0.00	0.8200		
DMA1DA01A11DA15210	8243A	0.954	MT-CO2	0.0000	E220K	0.00	0.7300		0.0008
DMA28501A11DA16V10	10233A	0.935	MT-ND3	0.0000	A59T	0.00	0.7700		0.0004
DMA28C01A11DA16V10	3380A	0.976	MT-ND1	0.0003	R25Q	0.00	0.8800	MELAS	0.0004
DMA28G01A11DA16V10	7623T	0.967	MT-CO2	0.0000	T13I	0.00	0.7700	LHON	
G4629401A11D180610	4222C	0.95	MT-ND1	0.0000	S306P	0.00	0.7800		
G4629501A11D171910	6744A	0.792	MT-CO1	0.0000	G281S	0.00	0.7700		
G4631501A11D171910	11711A	0.792	MT-ND4	0.0003	A318T	0.00	0.7800		0.0037
G4632001A11D171910	9384A	0.893	MT-CO3	0.0000	D60N	0.00	0.7800		0.0008
G4658801A11D177110	4004C	0.768	MT-ND1	0.0000	M233T	0.00	0.4800		

Tumor-specific variants recognized on COAD-derived mtDNAs and prioritized according to the established criteria. Sample identifier (sample), heteroplasmic fraction (HF), mtDNA locus (locus), site-specific nucleotide variability (Nt Var), amino acid change and variability (AA change and AA Var, respectively), Disease Score, annotations from Mitomap (Lott et al. 2013) and frequencies in 1000 genomes [as implemented in (Calabrese et al. 2014)] are associated with each variant allele. For full tumor-specific variants in COAD samples, see Supplementary Table 3—TumorSpecific

*HCM* hypertrophic cardiomyopathy, *MELAS* mitochondrial encephalomyopathy, lactic acidosis, and stroke-like episodes

### Germline variants in COAD samples

The process of prioritization was then applied on the dataset of variants shared between matched blood-tumor pairs to identify potentially pathogenic germline candidates that may represent predisposing variants. The filtering steps applied on the 716 germline variants, with an average of 64 variants per sample, identified in all COAD individuals (Supplementary Table 3—Germline), are shown in Fig. 3b. 76 % of total variants was recognized against rCRS, RSRS and MCHS while 27 % occurred in non-haplogroup-defining sites. 8 % of fully annotated variants was then filtered as featuring variability value below the established NVC, 9 non-synonymous variants remained. Among these, 7 showed a disease score above the DST. To further trim down the number of the suitable germline candidates for subsequent functional analyses, these were filtered for HF levels above 0.8, leading to prioritization of 4 variants (with confidence interval (CI) overlapping in both tumor and blood samples), corresponding to 0.56 % of total (Table 5). None of the prioritized germline variants had been previously annotated in Mitomap and may, therefore, deserve further investigation. The total number of prioritized variants on the strength of the criteria here proposed in the 90 COAD samples included 25 variants. Among these, 84 % of variants resulted to be tumor specific. It is worth noting that 266 variants were exclusively identified in 53/90 blood samples (Fig. 3; Supplementary Table 3—BloodSpecific), with an average of 3 variants per sample. Two hundred and sixty-one out of 266 variants were recognized against rCRS, RSRS and MCHS, all mapping on non-haplogroup-defining sites. Among these, 206 variants featured variability values lower than or equal to NVC, including 8 premature stop codons and 89 non-synonymous variants. Seventy-one out of 89 (27 % of the total variants) showed a disease score higher than or equal to DST and low HF levels ranging between 0.006 and 0.156. Thus, the 71 blood-specific non-synonymous variants (bold type in Supplementary Table 3—BloodSpecific) were not prioritized as potentially pathogenic.

**Table 5:** List of prioritized germline non-synonymous variants in COAD samples

Individual ID	Variant allele	HF blood/tumor	Locus	Nt Var	AA change	AA Var	Disease score	1000 genomes
G4629410A	9447C	1/1	MT-CO3	0.0000	Y81H	0.0155	0.7772	0.0012
AY619710A	9106G	1/0.88	MT-ATP6	0.0004	T194A	0.0009	0.6682	
F4680710A	8861T	1/1	MT-ATP6	0.0017	T112 M	0.0276	0.5456	
A6565710A	15434T	0.98/0.99	MT-CYB	0.0015	L230F	0.0016	0.7115	0.0008

Non-synonymous germline variants recognized on COAD-derived mtDNAs and prioritized according to the established criteria. Sample identifier (individual ID), heteroplasmic fraction in blood and tumor tissues (HF blood/tumor), mtDNA locus (locus), site-specific nucleotide variability (Nt Var), amino acid change and variability (AA change and AA Var, respectively), Disease Score, somatic (+) or germline (−) nature (‘tumor-specific/germline’) of variants and frequencies in 1000 Genomes [as implemented in (Calabrese et al. 2014)] are associated with each variant allele. For full germline variants in COAD samples, see Supplementary Table 3—Germline

The lists of prioritized variants of COAD datasets obtained by removing the haplogroup-filtering step, did not differ from those obtained performing the whole multi-step workflow, proving that there are not variants that may potentially affect function of gene/protein among the haplogroup-defining ones, underlying the good performance of our approach on cancer dataset.

## Discussion

The assessment of the role of mitochondrial variants in the onset and/or progression of human diseases and cancer is a difficult task. It requires robust and statistically reliable methods to support clinicians in the functional investigation of variants potentially affecting protein function. The question of determining the potential pathogenicity of mtDNA variants is in fact a matter of debate among clinicians (McCormick et al. 2013). Because of the peculiarities of mitochondrial polyplasmic genetics, assignment of pathogenicity should take into account the degree of heteroplasmy, the haplogroup background, and even environmental factors (Wallace et al. 2003). Several methods have been recently published to meet these goals, such as mit-o-matic (Vellarikkal et al. 2015), MitImpact (Castellana et al. 2015) and MtSNPscore (Bhardwaj et al. 2009). Here, we contribute with a robust statistical approach based on the introduction of two thresholds, NVC and DST. NVC threshold is calculated from the updated site-specific nucleotide variability values in a large dataset of complete healthy genomes available in HmtDB database, whose sequence quality was supported by a correct haplogroup prediction (see “Materials and methods”); DST is a statistically estimated robust threshold based on the pathogenicity predictions estimated by MutPred (Li et al. 2009), HumDiv- and HumVar-trained PolyPhen-2 models (Adzhubei et al. 2013), SNPs&GO, PhD-SNP (Capriotti et al. 2013), and PANTHER (Thomas and Kejariwal 2004), combined with the nucleotide variability distribution in the same HmtDB healthy dataset. To date, the information on biochemical and structural features of mitochondrial respiratory chain proteins are not so rich as for nuclear ones. Accordingly, all the in silico pathogenicity predictions may not be reliable and hence they should be considered with caution, since the determinants of pathogenicity for an mtDNA variant are unclear. The integration of pathogenicity predictions with the functional assays is an essential and strongly recommended step to consolidate the validation for pathogenicity of an mtDNA variant. The prioritization workflow is fully implemented in MToolBox (Calabrese et al. 2014) thus contributing to highlight mitochondrial DNA variants as suitable candidates for subsequent functional analyses in clinical studies—an issue which has often revealed to be problematic in analyses seeking correlations between mtDNA genotypes and disease phenotypes.

The in silico prioritization criteria here developed were established on the assumption that rare variants may more likely affect the gene/protein function than polymorphic variants (Bannwarth et al. 2013). In this context, we used the Macro Haplogroup Consensus Sequences to move the fixed evolutionary variants to the background and highlight rare ones. These rare variants may be characterized by nucleotide variability values below the established cutoff, while their potential functional impact, particularly for non-synonymous variants, may be assessed from the disease score definition. Variants mapping on the non protein-coding regions may be also prioritized by taking into account the filtering against the three references and the nucleotide variability cutoff (NVC) only. Pathogenicity data regarding tRNAs and rRNAs variants will be soon integrated in the proposed pipeline.

The application of the NVC and DST on the dataset of all possible non-synonymous variants (patho_table, https://sourceforge.net/projects/mtoolbox/) suggested that the loci *MT*-*ATP6*, *MT*-*ATP8* and *MT*-*CYB* harbor the variants featuring the highest nucleotide variability values and number of sites. Accordingly, these genes seem to be the least conserved regions of the human mtDNA and the most prone to harbor non-synonymous variants (Supplementary Fig. 4a), as previously reported in Mitomap. On the other hand, the distribution of disease scores related to all the potentially pathogenic variants occurring in the thirteen protein-coding regions did not show any gene-specific peculiarity (Supplementary Fig. 4b).

We applied the prioritization workflow to LHON, COAD and ovarian cancer sample sets to validate the robustness of our approach.

The majority of the causative mutations expected (8 out of 11 LHON-causing mutations) on the LHON dataset was recognized as affecting function by the application of the whole stepwise prioritization workflow with the exception of the mutations m.3700G>A (*MT*-*ND1*), m.14502T>C (*MT*-*ND6*) and m.14484T>C (*MT*-*ND6*) (Supplementary Table 1). These mutations were kept out by the application of chosen variability and/or disease score thresholds but were retained by the other filtering steps. Specifically, the mutation m.14484T>C was found in healthy individuals (0.11 % of total mtDNA genomes stored in HmtDB) despite the high DS suggestive of its potential functional impact. Such mutation was formerly considered a non-pathogenic variant, resulting in a conservative change into an amino acid with similar physiochemical properties (p.M64 V) (Mackey and Howell 1992) and found in population surveys without expressing any pathological phenotype (Achilli et al. 2012). However, it was previously assumed that it may exert a pathogenic potential if found in association with the haplogroups J and I (Achilli et al. 2012). 12 % of LHON-derived sequences here analyzed harbored this mutation; among these, only two belong to haplogroup J1 whereas most of the genomes belong to the macro-haplogroup M (26 % of total). The mutation m.3700G>A, although absent from the healthy population and for this reason more likely to have a functional impact, was suggested to be benign by the disease score. It may be due to half of pathogenicity predictors used to calculate the disease score, suggesting this mutation as benign (Supplementary Table 1—AllVariants sheet). This points out that pathogenicity predictions should always be treated with caution and that functional validation of variants is of paramount importance to clarify their role in contributing to phenotype, especially in the context of specific haplogroups and upon taking into account that penetrance is a cogent clinical issue particularly in canonical mitochondriopathies such as LHON. The mutation m.14502T>C, excluded since predicted as benign and found in the healthy population, is reported in the literature in sporadic LHON cases and/or in combination with other primary LHON mutations (Zhao et al. 2009; Zhang et al. 2010). In addition, our study suggested a pool of new variants not previously associated with LHON warranting further investigation with the aim to recognize novel causative mutations which could be added in the list of “top 14” LHON-specific mutations (Brandon et al. 2005). Finally, two additional variants were prioritized removing the haplogroup-filtering step. This result does not imply that our prioritization process may give false negatives because the output of the pipeline reports the entire list of variants with the prioritized ones at the top. The user is free to filter the variants according to his preferred criteria. The system simply suggests and reports the thresholds that may facilitate the selection of functional important variants.

With respect to cancer, it needs to be underlined that our workflow may provide an additional advantage, namely the efficient selection of potentially affecting function variants that are candidates for being somatic that only transformed cells may withstand, in the context of a deregulated cell metabolism. For too long, associations between cancer types and mtDNA variants have been reported without verifying whether they were somatic or germline, in which latter case they may be speculated to be predisposing to transformation. Even further, many polymorphisms have been classified in the past years as cancer-associated mtDNA mutations (Máximo and Sobrinho-Simões 2000; Setiawan et al. 2008), a risky statement likely driven by the lack of well-curated databases and of a commonly agreed protocol to define such associations. The high frequency of mtDNA variants that is often detected in cancer samples may also be a great obstacle to select few candidates to bring forward to functional studies, with the aim to assess in which way they may impact metabolism. This is a key step that this workflow intends to simplify, as we have shown in both COAD and ovarian cancer sample sets by verifying the somatic nature of prioritized variants. However, we have here shown that our criteria are not too stringent, as they allow inclusion of a few variants that were not found to be somatic. Although this finding may somewhat decrease the performing index of our workflow in highlighting specifically cancer-associated mutation, it may on the other hand permit to select variants that may still affect the gene/protein function, even though not associated to the patient’s disease.

In this paper, we successfully demonstrate that the prioritization of human mtDNA variants based on the workflow here proposed is able to recognize potential affecting function variants. Compared to the existing pipelines, capable of annotating mitochondrial variations from next-generation datasets exclusively (mit-o-matic, Vellarikkal et al. 2015), or extract information from lists of variants or FASTA sequences exclusively (MtSNPscore, Bhardwaj et al. 2009), our prioritization workflow, currently being implemented in MToolBox, appears to be more flexible allowing a functional annotation of variants on large datasets from both next-generation (in BAM, SAM, FASTQ format) and Sanger sequencing (in FASTA format) data.

Finally, the recent publication of MitImpact (Castellana et al. 2015) reports a database of the entire dataset of non-synonymous human mtDNA variants annotated with nucleotide variability available through the HmtDB resource and pathogenicity scores estimated by applying the same predictors implemented in MToolBox, with three additional ones. The estimation of the level of concordance among predictions is provided through two alternative “uniformity scores”, similar to our percentage of concordance, previously implemented in MToolBox and now replaced by the disease scores here reported. The different type of inputs required by mit-o-matic, MitImpact and MtSNPscore did not allow to report here the results of a quantitative comparison with MToolBox. Those results may be roughly comparable according to predicted pathogenicity of the considered variants.

In conclusion, our prioritization workflow based on (1) the use of MHCSs together with RSRS and rCRS, to filter out variants fixed during evolution; (2) the availability of the NV scores obtained through the HmtDB annotated genomes and (3) the implementation of the NVC and DST, reducing the large amount of variants from both NGS and conventional Sanger technologies, offers one of the most complete tools to guide clinicians in selecting the non-synonymous mtDNA variants with a potential functional impact. However, functional assays are strongly required to confirm the pathogenicity of all mtDNA variants prioritized by this workflow, as well as by any automated method, with the aim to establish their exact role and involvement in disease phenotypes.

## Electronic supplementary material

Supplementary material 1 (PDF 1810 kb)

Supplementary material 2 (XLS 2225 kb)

Supplementary material 3 (XLS 325 kb)

Supplementary material 4 (XLS 1978 kb)

Supplementary material 5 (XLS 567 kb)
