# Supplementary material for: A multi-parametric workflow for the prioritization of mitochondrial DNA variants of clinical interest
Source: Hum Genet. 2015 Nov 30;135:121–36. doi: 10.1007/s00439-015-1615-9 (PMC4698288; doi:10.1007/s00439-015-1615-9)
Supplement: Supplementary file 1 — Supplementary material 1 (PDF 1810 kb) [file 439_2015_1615_MOESM1_ESM.pdf]

Electronic Supplementary Material

A multi-parametric workflow for the prioritization of mitochondrial DNA variants of clinical interest

Journal: Human Genetics

Mariangela Santorsola[1,2], Claudia Calabrese[3], Giulia Girolimetti[3], Maria Angela Diroma[1], Giuseppe Gasparre[3§], Marcella Attimonelli[1§*]

[§]Co-last authors

[1]Department of Biosciences, Biotechnologies and Biopharmaceutics, University of Bari, Via E.Orabona 4, 70126 Bari, Italy

[2]Department of Science and Technologies, University of Sannio, Via Port'Arsa 11, 82100 Benevento, Italy

[3]Department of Medical and Surgical Sciences, Medical Genetics, University of Bologna Medical School, via Massarenti 9, 40138, Bologna, Italy

Marcella Attimonelli

Department of Biosciences, Biotechnologies and Biopharmaceutics, University of Bari -Italy

Via E.Orabona 4, 70126 Bari (IT)

Tel +390805442399

Fax +390805443317

e-mail marcella.attimonelli@uniba.it

Supplementary Table 1 LHON-derived samples analysis

Working folder lists haplogroup predictions, geographic origins, Genbank and HmtDB identifiers (Genbank ID and HmtDB ID) of 125 LHON-derived samples (SampleData sheet) and the overall list of variants sorted according to prioritization criteria, with related functional annotations obtained by MToolBox (AllVariants sheet). For each sample (Sample) and for each variant (Variant Allele) the functional annotation reports:

-pathogenicity predictions and probabilities (if the variant is non-synonymous), estimated using MutPred (Li et al. 2009), HumDiv- and HumVar-trained PolyPhen-2 (Adzhubei et al., 2013), SNPs&GO, PhD-SNP (Capriotti et al. 2013), and PANTHER (Thomas and Kejariwal 2004) algorithms and the percentage of these models predicting the variant as 'Disease', 'Neutral' or 'Unclassified';

-the Disease Score for each non-synonymous variant;

-annotations from MITOMAP (Lott et al., 2013), including homoplasmy/heteroplasmy data;

-link to OMIM (http://omim.org) and Mamit-tRNA (Pütz et al., 2007) web resources;

-the allele frequency in the 1000 Genomes (Genomes Project et al., 2012) samples.

Variability values lower then or equal to the Nucleotide Variability Cut-off, NVC, and disease scores higher than or equal to the Disease Score Threshold, DST, are in bold type. The command line used to run MToolBox on this dataset is: MToolBox.sh -i fasta.

Supplementary Table 2 Ovarian cancer analysis

Working folder lists identifiers, HmtDB ID and haplogroup predictions of ovarian cancer samples (SampleData sheet). Overall list of variants sorted according to prioritization criteria with related functional annotations obtained by MToolBox and information about the somatic or germline nature ('tumor-specific/germline') of the prioritized variants are reported (AllVariants sheet). For each sample and for each variant (Variant Allele) the functional annotation reports:

-pathogenicity predictions and probabilities (if the variant is non-synonymous), estimated using MutPred (Li et al. 2009), HumDiv- and HumVar-trained PolyPhen-2 (Adzhubei et al., 2013), SNPs&GO, PhD-SNP (Capriotti et al. 2013), and PANTHER (Thomas and Kejariwal 2004) algorithms and the percentage of these models predicting the variant as 'Disease', 'Neutral' or 'Unclassified';

-the Disease Score for each non-synonymous variant;

-annotations from MITOMAP (Lott et al., 2013), including homoplasmy/heteroplasmy data;

-link to OMIM (http://omim.org) and Mamit-tRNA (Pütz et al., 2007) web resources;

-the allele frequency in the 1000 Genomes (Genomes Project et al., 2012) samples.

Variability values lower then or equal to the Nucleotide Variability Cut-off, NVC, and disease scores higher than or equal to the Disease Score Threshold, DST, are in bold type. Synonymous variants recognized as germline are in italics. The command line used to run MToolBox on this dataset is: MToolBox.sh -i fasta.

Supplementary Table 3 COAD-derived samples analysis

Working folder lists subject identifiers (Blood and Tumor fields) and associated haplogroup predictions, mean coverage depth and percentage of assembled bases for each mtDNA genome from tumor and blood samples (TumorSamples and BloodSamples sheets). Overall list of germline, tumor-specific and blood-specific variants sorted according to prioritization criteria and related functional annotations obtained by MToolBox are also reported (Germline, TumorSpecific and BloodSpecific sheets). For each sample and for each variant (Variant Allele) the functional annotation reports:

-pathogenicity predictions and probabilities (if the variant is non-synonymous), estimated using MutPred (Li et al. 2009), HumDiv- and HumVar-trained PolyPhen-2 (Adzhubei et al., 2013), SNPs&GO, PhD-SNP (Capriotti et al. 2013), and PANTHER (Thomas and Kejariwal 2004) algorithms and the percentage of these models predicting the variant as ‘Disease’, ‘Neutral’ or ‘Unclassified’;

-the Disease Score for each non-synonymous variant;

-annotations from MITOMAP (Lott et al., 2013), including homoplasmy/heteroplasmy data;

-link to OMIM (http://omim.org) and Mamit-tRNA (Pütz et al., 2007) web resources;

-the allele frequency in the 1000 Genomes (Genomes Project et al., 2012) samples.

Heteroplasmy levels, the related confidence interval (CI_lower;CI_upper) in tumor/blood samples (HF Tumor and HF Blood fields), and information about overlapping confidence intervals (HF Overlapping) in both tissues are also reported for each germline variant (Germline sheet). Variability values lower then or equal to the Nucleotide Variability Cut-off, NVC, and disease scores higher than or equal to the Disease Score Threshold, DST, are in bold type. The command line used to run MToolBox on bam files: MToolBox.sh -i bam -M -I -a "-t 10".

Supplementary Table 4 Disease score data

Working folder lists the non-synonymous variants described in the text previously validated as deleterious included in the training dataset for the estimation of the disease score (TrainingDataset sheet). The weight values assigned to each pathogenicity prediction method and the keys are reported in the Keys sheet.

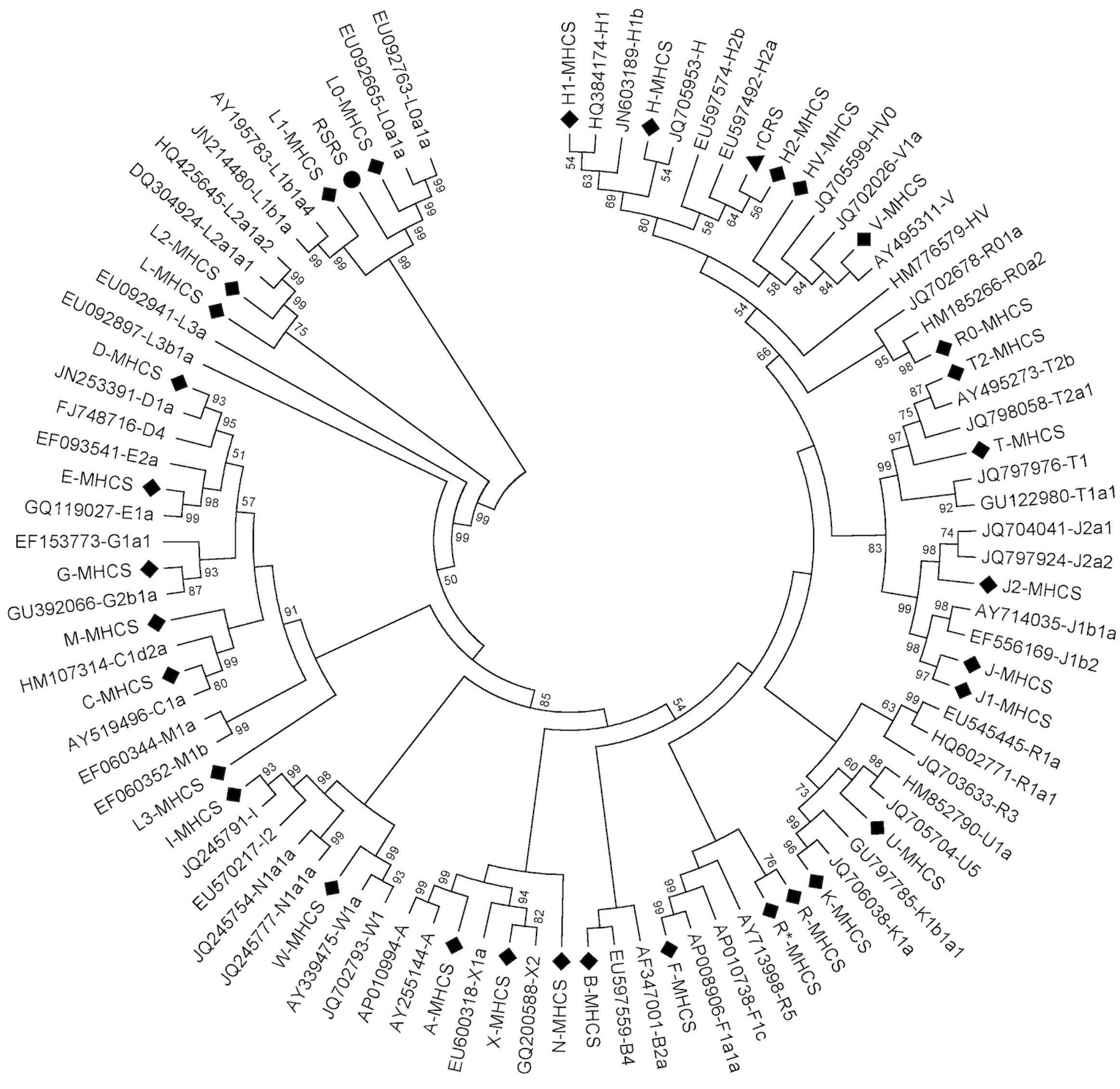

Supplementary Figure 1

Phylogenetic relationships among virtual Macro Haplogroup Consensus Sequences (MHCS) and two real mitochondrial sequences for each haplogroup (Brandon et al. 2005). Maximum Likelihood method based on Jukes-Cantor substitution model (Jukes and Cantor 1969) was applied to 93 nucleotide sequences, aligned with MAFFT online version (Katoh and Standley 2013). Figure shows the tree with the highest log likelihood (-30144.1188). Black squares: haplogroup-specific MHCS; black triangle: rCRS; black circle: RSRS. Tree building also involved bootstrapping repeated 500 times to generate majority consensus tree. Bootstrap values (>50 and mainly near 100) shown next to branches, supporting tree robustness.

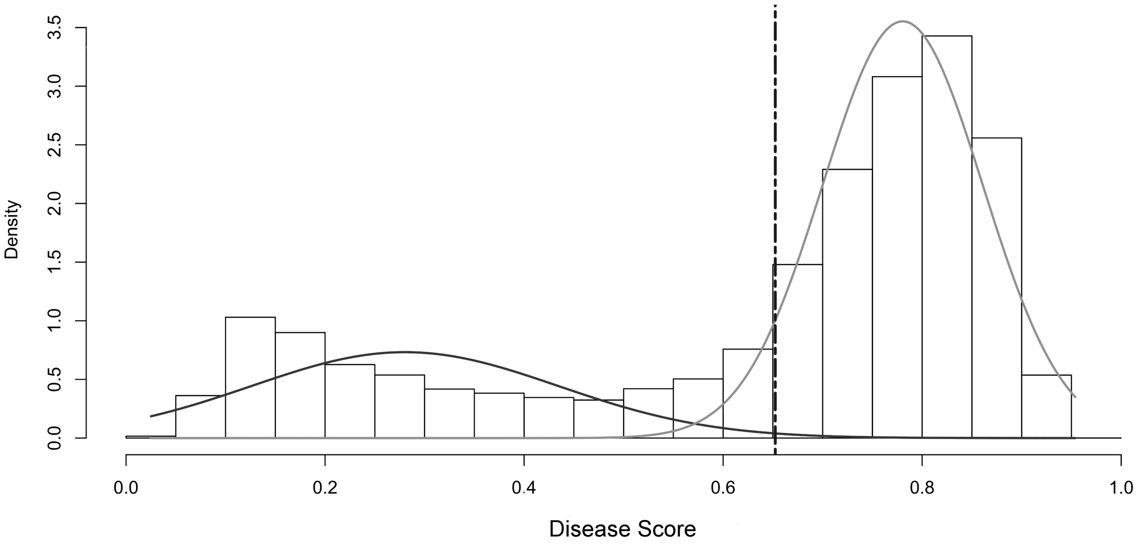

Supplementary Figure 2

The histogram graphs the bimodal distribution of disease scores associated with all possible 24,195 non-synonymous variants reported in the patho_table provided with MToolBox. The solid lines indicate the two gaussian components of the mixture model (McLachlan and Peel 2000). The vertical dashed line is drawn at the selected Disease Score Threshold, DST =0.652, defined as described in the Results section. This DST should underestimate the potential functional role of a variant, since it was defined from the DS distribution of non-synonymous variant dataset including variants never observed in mtDNA genomes from both healthy and diseased individuals.

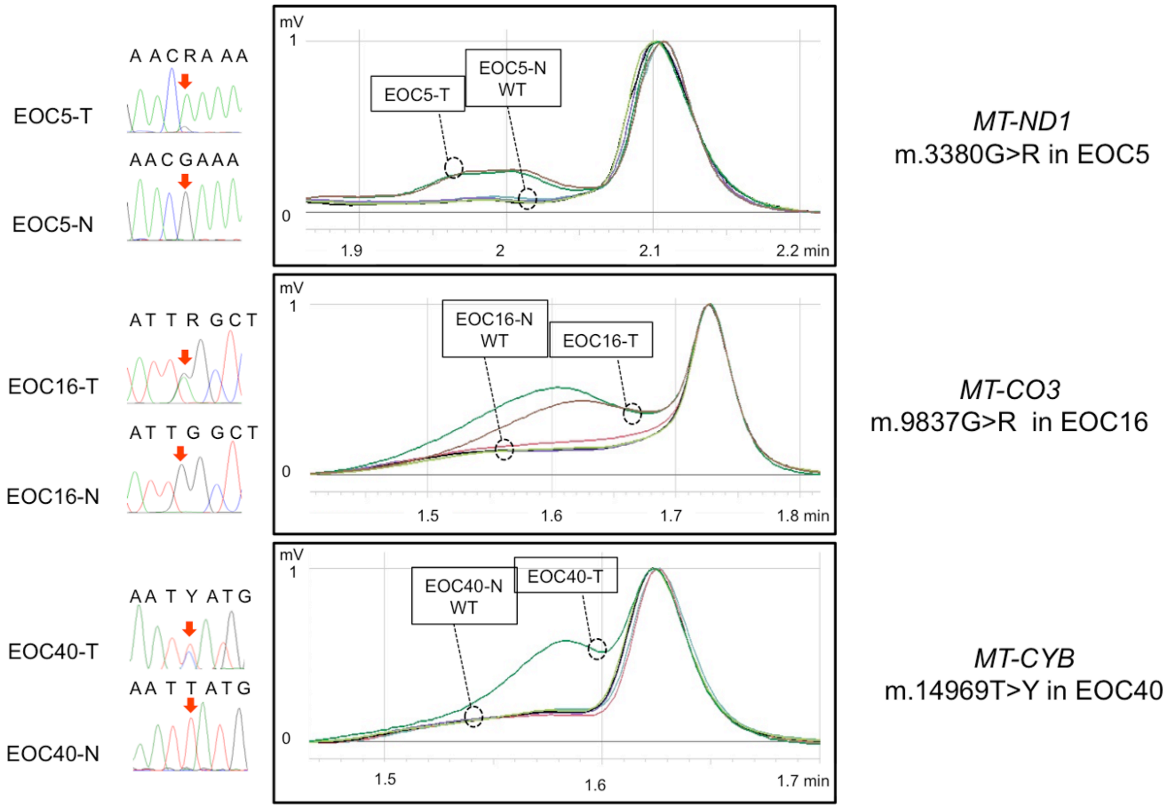

Supplementary Figure 3

Electropherogram and dHPLC analysis of the heteroplasmic m.3380G>R in *MT-ND1* in EOC5, m.9837G>R in *MT-CO3* in EOC16, m.14969T>Y in *MT-CYB* in EOC40 mutations. Homo and heteroduplexes are distinguished based on different retention times. Two elution curves for ovarian cancer tissue (heteroduplex and homoduplex) and a single elution curve for non-tumor tissue and two wild-type controls are present. Wild-type (pink and purple), non-tumor tissue (black and light blue), ovarian tumor (green and brown). T, Ovarian cancer tissue; N, Non tumor tissue; WT, wild type.

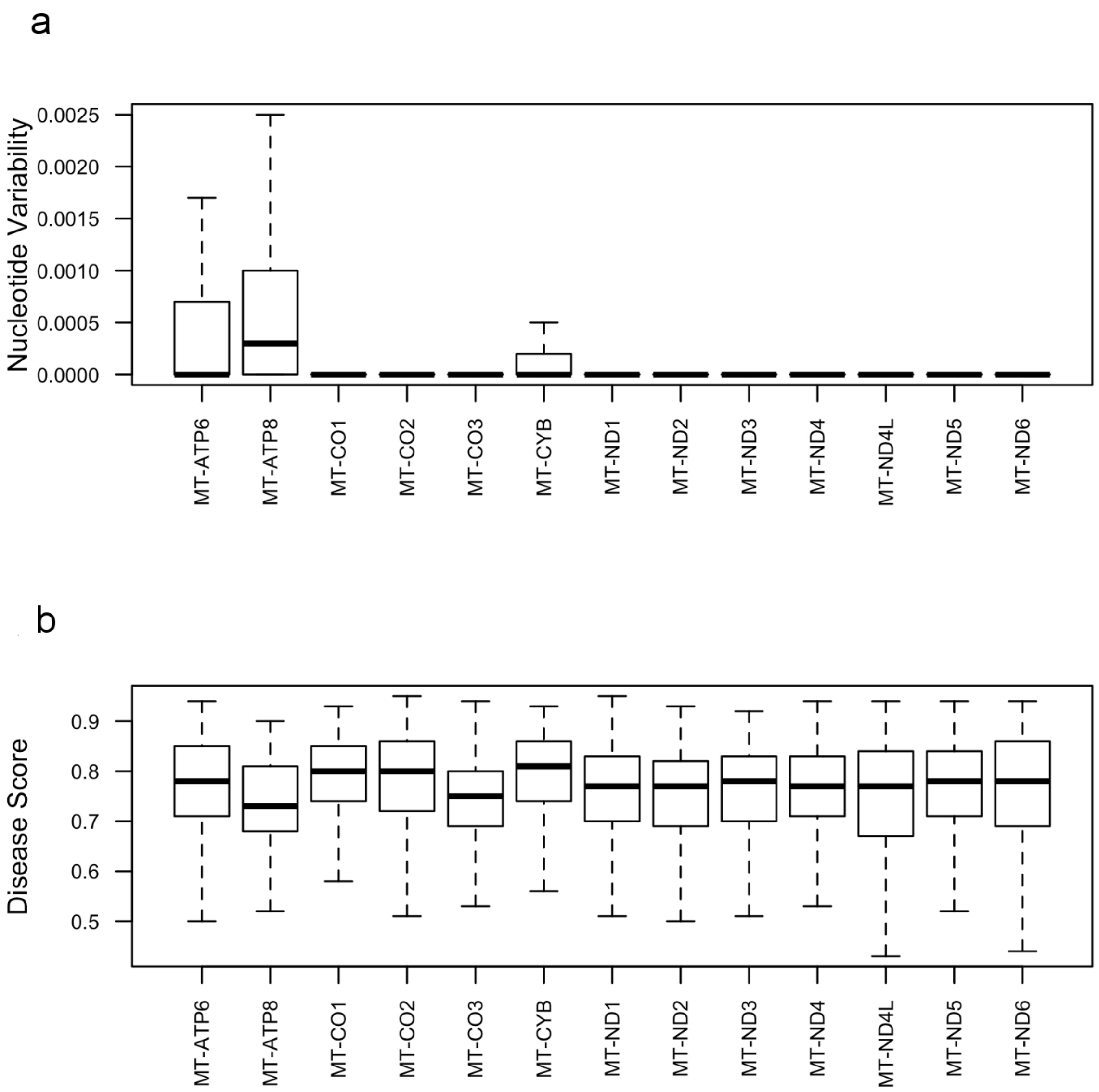

Supplementary Figure 4

A) Box-plot diagram showing the distribution of nucleotide variability values associated to all possible non-synonymous variants (Calabrese et al. 2014) selected as potentially pathogenic on the strength of prioritization criteria, in each mitochondrial protein-coding gene. B) Box-plot diagram showing the distribution of the disease scores associated to all possible non-synonymous variants (Calabrese et al. 2014) selected as potentially pathogenic on the strength of prioritization criteria, in each mitochondrial protein-coding gene.
